# Ten-year outcomes of repeat keratoplasty for optical indications

**DOI:** 10.3389/fmed.2024.1503333

**Published:** 2025-01-22

**Authors:** Victoria Grace Dimacali, Hon Shing Ong, Stephanie Shuang Lang, Hla Myint Htoon, Howard Cajucom-Uy, Hui Chen Charmaine Chai, Marcus Ang, Anshu Arundhati, Jodhbir S. Mehta

**Affiliations:** ^1^Corneal and External Eye Disease Department, Singapore National Eye Centre, Singapore, Singapore; ^2^Tissue Engineering and Cell Therapy Group, Singapore Eye Research Institute, Singapore, Singapore; ^3^Ophthalmology and Visual Sciences Academic Clinical Program, Duke-NUS Medical School, Singapore, Singapore; ^4^Singapore Eye Bank, Singapore National Eye Centre, Singapore, Singapore; ^5^Singapore Eye Research Institute, Singapore, Singapore; ^6^Yong Loo Lin School of Medicine, National University of Singapore, Singapore, Singapore

**Keywords:** corneal transplant, repeat keratoplasty, regraft, graft failure, graft survival

## Abstract

**Aim:**

To analyze the clinical outcomes of repeat keratoplasties following a failed primary optical keratoplasty in an Asian population.

**Methods:**

In this prospective cohort study, clinical data of consecutive patients who had repeat keratoplasty at Singapore National Eye Centre from 2007 to 2020 were recorded from our ongoing Singapore Corneal Transplant Registry.

**Results:**

A total of 284 first regrafts (181 Descemet stripping automated endothelial keratoplasty (EK), 63 penetrating keratoplasty (PK), 21 Descemet membrane endothelial keratoplasty, and 19 deep anterior lamellar keratoplasty (DALK)) were analyzed. Graft rejection (38.4%) and late endothelial failure (15.8%) were the main causes of initial graft failure. PK/EK and EK/EK had better one-year postoperative best corrected visual acuity compared to PK/PK (*p* = 0.006 and *p* < 0.001 respectively). Kaplan–Meier 10-year cumulative regraft survival probabilities were 69.2% for PK/EK, 52.8% for EK/EK, and 43.1% for PK/PK. Regraft survival was 100% for DALK/PK, DALK/DALK, and DALK/EK at three to 5 years. Log-rank test showed higher survival of PK/EK compared to PK/PK (*p* = 0.002) and EK/PK (*p* = 0.009), and of EK/EK compared to PK/PK (*p* = 0.003) and EK/PK (*p* = 0.005). High-risk regrafts had significantly lower 10-year survival probabilities compared to non-high-risk regrafts (*p* = 0.045). Cox multiple regression analysis showed male gender (*p* = 0.023), PK regraft (*p* = 0.003), regraft rejection (p = 0.003), and initial graft indications of pseudophakic bullous keratopathy (*p* = 0.005) and aphakic bullous keratopathy (*p* = 0.004) to be risk factors for regraft failure, while longer time to regraft was associated with decreased risk of failure (*p* = 0.013).

**Conclusion:**

Performing EK for failed optical PK or EK significantly improved regraft survival compared to repeat PK. Regrafts performed for failed initial DALK grafts did well regardless of type.

## 1 Introduction

Corneal transplantation, the most common transplantation procedure, has significantly evolved since the first successful human penetrating keratoplasty (PK) performed in 1905 (1, 2). With increasing numbers of keratoplasties being performed worldwide, the result is a rising incidence of graft failure as an indication for repeat keratoplasty (3, 4). Previously, the only option for a failed PK was a repeat PK. Through more advanced techniques of lamellar keratoplasty, it is now possible to selectively replace only the affected corneal layer(s). Descemet stripping automated endothelial keratoplasty (DSAEK) or Descemet membrane endothelial keratoplasty (DMEK), instead of repeat PK, can be done for PK with endothelial failure but without stromal scarring (5–8). Endothelial keratoplasty (EK) is also now an option for deep anterior lamellar keratoplasty (DALK) with failed host Descemet membrane-endothelium complex (1). Conversely, DALK can be performed for eyes which developed stromal scarring after DSAEK where functioning endothelium still exists. These effectively decrease the alloantigen load transplanted to the recipient eye, thought to lead to better graft survival and lower risk of immunological rejection (9).

Graft failure has now been reported by some centers to be one of the leading indications for corneal transplantations (4, 10). However, overall regraft survival rates as well as visual outcomes of repeat grafts have been reported to be worse compared to primary grafts, even if the original indication was a low-risk one (5–13). This may be due to risk factors from the initial surgery such as inflammation, corneal neovascularization, peripheral anterior synechiae, glaucoma, and poorer ocular surface (5, 14–16). A previously failed graft also increases the risk of rejection of succeeding grafts since there is more efficient immunization against donor antigens, although the exact mechanism is still not well understood (14).

Recent studies comparing the survival of regrafts according to technique have indicated varying results, with few having long term follow-up or a large number of patients (6–9, 12, 13). Through a meta-analysis of four studies, Wang et al. reported a lower risk of graft rejection in EK for a failed PK, compared to a repeat PK (*p* = 0.007) (4). No differences in graft survival and visual acuity however were seen between the two groups (*p* = 0.81 to 0.97), although the studies between themselves had contrasting results. Data available on repeat anterior lamellar keratoplasty (ALK) is also limited (15).

Using corneal transplant registry data, we aimed to investigate the indications and methods of repeat corneal transplantations, and to determine the clinical outcomes of repeat keratoplasties of eyes with failed optical PK, EK, or DALK.

## 2 Materials and methods

Clinical data of patients who undergo corneal transplantation at the Singapore National Eye Centre (SNEC) are prospectively recorded in the Singapore Corneal Transplant Registry (SCTR) during periodic follow-ups. The criteria used by the Singapore Eye Bank for optical donor tissue for PK, DSAEK, DMEK, and DALK are shown in Supplementary Table S1. For this study, the records of patients with a previous failed primary optical graft who underwent their first optical regraft, defined as a regraft performed for optical indications, from 2007 to 2020 were retrieved from the SCTR and analyzed. In order to avoid the effect of worsening prognosis with increasing regraft numbers, we limited our study to the first repeat optical keratoplasties (i.e., second grafts) (6). Included were cases of primary graft failure, late endothelial failure, and failure secondary to glaucoma, infection, persistent ocular surface disease, trauma, graft rupture, and subsequent surgery. Primary graft failure was defined as persistence of corneal edema until the sixth postoperative week, in the absence of any operative or postoperative complication or underlying recipient condition. Late endothelial failure refers to later failure occurring without evidence of rejection such as a Khodadoust line. The following were excluded: tectonic or therapeutic first or second grafts, patients less than 16 years old at the time of first regraft, and second eyes of patients who have bilateral first optical regrafts. Minimum follow-up was 6 months unless the regraft had irreversibly failed before then. This study was approved by the Singapore Health Services Centralized Institutional Review Board and adhered to the principles of the Declaration of Helsinki.

Patient demographics, first and second graft types, indications, time from first graft failure diagnosis to repeat surgery, regraft complications including graft failure, and duration of follow-up were analyzed. Snellen best corrected visual acuity (BCVA) and regraft success at 1 year after surgery were also assessed. Success was defined as a clear graft, while survival of regrafts was the time from the date of repeat transplantation to the date at which the graft was assessed by a corneal specialist to have had irreversibly lost its clarity.

### 2.1 Surgical technique

All transplants were performed by 10 experienced corneal surgeons using standardized techniques for PK, DSAEK, DMEK and DALK previously described (7, 17). Limited Descemet membrane stripping was done in cases of PK/DMEK while no stripping was done in PK/DSAEK. Most DSAEK cases were accomplished using a pull-through technique with a Coronet DSAEK EndoGlide (Network Medical, United Kingdom), while the rest utilized a push-through technique with a Sheets glide. DMEK grafts were inserted using an endothelium-in pull-through or an endothelium-out injection technique (18). Anwar big-bubble or manual dissection was performed for DALK (19).

### 2.2 Postoperative management

A standard postoperative therapeutic regimen previously described by our group composed of a steroid and an antibiotic was given to all patients (7). For PK and EK regrafts, levofloxacin 0.5% and prednisolone acetate 1% eye drops were started at one drop every 3 h for the first month, then 4 times a day for 2 months. The steroid was then tapered by one drop every 3 months until one drop a day at 1 year was reached and maintained indefinitely. In cases of DALK regrafts, dexamethasone was given and tapered off to discontinue by 6 months (20).

High-risk cases in this study were defined as having one or more of the following factors, in addition to having a regraft: superficial or deep vascularization in one or more quadrants, glaucoma or increased IOP, active inflammation, ocular surface disease, lid disease, history of ocular trauma, large (≥9 mm) primary and/or repeat graft, and the presence of anterior synechiae. Regrafts without any other additional risk factor were defined as non-high-risk grafts for this study (although being themselves regrafts already makes them high-risk cases). Select high-risk cases were given additional immunosuppression, starting with topical ciclosporin 0.5% BD. Cases which needed additional short-term immunosuppression were also given oral prednisone at 10 mg/day for 1 month then 5 mg/day for 2 months; for long-term immunosuppression mycophenolate mofetil 250–500 mg q12 was given for at least 1 year.

### 2.3 Statistical analysis

Continuous, parametric variables were compared between groups using Kruskal-Wallis test, while categorical, non-parametric variables were compared using Chi-square test or Fisher’s exact test. Since the numbers of DMEK regrafts were too small in this study to generate meaningful analysis, DSAEK and DMEK regrafts were grouped together as EK regrafts. Preoperative and one-year postoperative BCVA among PK/PK, PK/EK, EK/EK, and EK/PK groups were compared using Mann–Whitney U test. Kaplan–Meier survival functions of the different combinations of primary grafts and regrafts, and of high-risk and non-high-risk regrafts were calculated using the Statistical Package for the Social Sciences (SPSS Statistics for Windows, Version 24.0. NY:IBM Corp.). Log-rank test was used to determine differences in survival between groups. Statistical significance was defined as a *p*-value of less than 0.05.

Univariate Cox regression analysis was performed to identify whether age, gender, race, and graft-related factors significantly influenced regraft survival. Risk factors with *p* < 0.05 were included in multivariate analysis.

## 3 Results

A total of 3,314 keratoplasties were performed over the study period, 791 (23.9%) of which were repeat keratoplasties. A total of 284 eyes which had a first optical regraft during this period and fulfilling the inclusion and exclusion criteria were identified. Baseline characteristics of the patients are shown in Table 1. The overall mean age at first regraft was 65.3 ± 14.6 years. There was a significant difference between the regraft groups with patients who underwent DALK as a regraft being younger (54.5 ± 20.9 years) than those who underwent PK (63.4 ± 14.8 years) or EK (67.0 ± 13.3 years) as a regraft (*p* = 0.017). Half of the patients in our study had pre-existing increased intraocular pressure (IOP) (50.7%). The incidence was twice as high among PK and EK regrafts (52.4 and 52.5%) than in DALK regrafts (26.3%), with the difference between groups trending toward significance (*p* = 0.089).

**Table 1:** Baseline demographics of the study population comparing PK, EK, and DALK regrafts (*n* = 284).

	All	PK as regraft	EK as regraft	DALK as regraft	*p*-value
No. of eyes	284 (100%)	63 (22.2%)	202 (71.1%)	19 (6.7%)	
Mean Age in years (SD) [Range]	65.3 (14.6) [17.5–95.6]	63.4 (14.8) [20.6–95.6]	67.0 (13.3) [17.5–94.4]	54.5 (20.9) [19.8–86.8]	0.017
Gender, *n* (%)					0.143
Male	164 (57.7)	39 (61.9)	118 (58.4)	7 (36.8)	
Female	120 (42.3)	24 (38.1)	84 (41.6)	12 (63.2)	
Race, *n* (%)					0.026
Chinese	217 (76.4)	47 (74.6)	159 (78.7)	11 (57.9)	
Malay	28 (9.9)	11 (17.5)	16 (7.9)	1 (5.3)	
Indian	20 (7.0)	2 (3.2)	14 (6.9)	4 (21.1)	
Others	19 (6.7)	3 (4.8)	13 (6.4)	3 (15.8)	
Increased IOP	144 (50.7%)	33 (52.4%)	106 (52.5%)	5 (26.3%)	0.089
Post-regraft complications^a^					
Increased IOP^b^	68 (23.9%)	25 (39.7%)	39 (19.3%)	4 (21.1%)	0.005
Regraft rejection	30 (10.6%)	15 (23.8%)	14 (6.9%)	1 (5.3%)	0.001
Microbial keratitis	5 (1.8%)	2 (3.2%)	2 (1.0%)	1 (5.3%)	0.108
Total complications	95 (33.5%)	37 (58.7%)	53 (26.2%)	5 (26.3%)	<0.001
Median follow-up time, months [Range]	26.2 [4.9–169.3]	25.1 [5.9–156.2]	26.1 [4.9–169.3]	31.7 [6.8–99.7]	0.974

PK, penetrating keratoplasty; EK, endothelial keratoplasty; DALK, deep anterior lamellar keratoplasty; SD, standard deviation; IOP, intraocular pressure. ^a^Not included are regraft detachments, of which three were recorded after EK from 2015 to 2020. ^b^Includes cases with pre-existing increased IOP. Percentages indicate the proportion of cases for that regraft type. A regraft may have had more than one recorded postoperative complication.

The median time from the first keratoplasty to repeat keratoplasty was 3.9 years (Supplementary Table S2). Initial PK grafts showed the longest median time interval between the two transplants (5.6 to 9.7 years), while initial DMEK grafts had the shortest median time from first graft to regraft (0.3 to 0.6 years). Initial PK grafts reached up to 26 years prior to regrafting.

The most common diagnoses for both initial PK and initial EK grafts were pseudophakic bullous keratopathy (PBK) (38.0%) and Fuchs endothelial dystrophy (FED) (17.6%) (Table 2). There were significantly more PBK and FED cases among initial EK than initial PK grafts (*p* = 0.002 and *p* = 0.019 respectively). Cases of failed PBK and FED represented a greater proportion of initial EK (49.3 and 24.0% respectively) than PK grafts (30.3 and 12.6% respectively), while cases of aphakic bullous keratopathy (ABK) were mostly from the failed PK group (10.9%). The most common indications for primary DALK were postinfectious scarring (31.6%) and keratoconus (26.3%).

**Table 2:** Indications for performing primary grafts and repeat grafts according to type of primary graft (*n* = 284).

Indications for first grafts	All Grafts	Initial PK grafts	Initial EK grafts	Initial DALK grafts
Pseudophakic bullous keratopathy	108 (38.0)	36 (30.3)	72 (49.3)	0 (0.0)
Fuchs endothelial dystrophy	50 (17.6)	15 (12.6)	35 (24.0)	0 (0.0)
Other causes of scarring/edema	40 (14.1)	13 (10.9)	24 (16.4)	3 (15.8)
Corneal dystrophy aside from FED and keratoconus	22 (7.7)	11 (9.2)	8 (5.5)	3 (15.8)
Keratoconus	17 (6.0)	12 (10.1)	0 (0.0)	5 (26.3)
Aphakic bullous keratopathy	16 (5.6)	13 (10.9)	3 (2.1)	0 (0.0)
Postinfectious scar/ thinning	14 (4.9)	8 (6.7)	0 (0.0)	6 (31.6)
Corneal injury	14 (4.9)	9 (7.6)	3 (2.1)	2 (10.5)
Congenital	2 (0.7)	2 (1.7)	0 (0.0)	0 (0.0)
CMV endotheliitis	1 (0.4)	0 (0.0)	1 (0.7)	0 (0.0)
Total	284 (100)	119 (100)	146 (100)	19 (100)
Causes of first graft failure				
Allograft rejection	109 (38.4)	58 (48.7)	48 (32.9)	3 (15.8)
Late endothelial failure	45 (15.8)	16 (13.4)	27 (18.5)	2 (10.5)
Primary graft failure	44 (15.5)	18 (15.1)	25 (17.1)	1 (5.3)
Pseudophakic bullous keratopathy	25 (8.8)	10 (8.4)	15 (10.3)	0 (0)
Recurrence of corneal dystrophy (including keratoconus)	17 (6.0)	5 (4.2)	10 (6.8)	2 (10.5)
Postinfectious scar/ thinning	15 (5.3)	4 (3.4)	8 (5.5)	3 (15.8)
Other causes of scarring/edema	11 (3.9)	2 (1.7)	6 (4.1)	3 (15.8)
Corneal injury	6 (2.1)	0 (0)	3 (2.1)	3 (15.8)
Increased IOP	4 (1.4)	3 (2.5)	1 (0.7)	0 (0)
Aphakic bullous keratopathy	3 (1.1)	2 (1.7)	1 (0.7)	0 (0)
Recurrence of primary disease	2 (0.7)	0 (0)	0 (0)	2 (10.5)
Subsequent surgery (corneal refractive surgery)	1 (0.4)	1 (0.8)	0 (0)	0 (0)
Others^a^	2 (0.7)	0 (0)	2 (1.4)	0 (0)
Total	284 (100)	119 (100)	146 (100)	19 (100)

PK, penetrating keratoplasty; EK, endothelial keratoplasty; DALK, deep anterior lamellar keratoplasty; FED, Fuchs endothelial dystrophy; CMV, cytomegalovirus. ^a^Cytomegalovirus endotheliitis, stromal scarring secondary to calcific band keratopathy. Numbers in parentheses indicate the proportion of cases for that graft type.

High-risk cases (177 (62.3%)) represented more than half of the study population. Additional immunosuppression was given to 41 patients (14.4%) as follows: topical ciclosporin 8.1% (23 patients), oral mycophenolate mofetil 2.5% (7 patients), oral ciclosporin 2.1% (6 patients), oral prednisone 1.1% (3 patients), topical ciclosporin and oral prednisone 0.7% (2 patients).

Overall, first graft failure in more than a third of the cases resulted from allograft rejection (38.4%); other more common causes of graft failure requiring regrafting were late endothelial failure (15.8%) and primary graft failure (15.5%) (Table 2). There were significantly more initial PK than initial EK grafts which failed due to rejection (48.7% of all initial PK grafts vs. 32.9% of all initial EK grafts, *p* = 0.009) while only 15.8% of failed initial DALK grafts were attributed to rejection (2.8% cases of initial graft failure from rejection). Similar proportions of initial PK and EK grafts were seen among cases of late endothelial failure (13.4% vs. 18.5% respectively, *p* = 0.267) and primary graft failure (15.1% vs. 17.1% respectively, *p* = 0.663).

### 3.1 Regraft techniques

The most common primary graft to undergo a regraft was EK (DSAEK 46.5% and DMEK 4.9%), followed by PK (41.9%) (Supplementary Table S3). Compared to their respective initial grafts, there was a greater proportion of EK regrafts (DSAEK 63.7% and DMEK 7.4%), while PK regrafts were fewer (22.2%); DALK regrafts remained the same (6.7%). The most frequently performed graft/regraft procedures were DSAEK/DSAEK (37.3%), PK/DSAEK (22.2%), and PK/PK (19.0%).

There were 47 cases (16.5%) which underwent at least one additional intraocular procedure during the first regraft (Supplementary Table S4); these were mostly phacoemulsification and intraocular lens (IOL) procedures. Cataract surgeries were more commonly combined with PK/PK or PK/EK, while IOL procedures were done more often with EK/EK.

### 3.2 Regraft complications

The most common complication after regrafting was increased IOP (pre-existing and *de novo*) in 23.9% of eyes (Table 1). This was more frequently seen among PK regrafts (39.7%) compared to DALK (21.1%) and EK regrafts (19.3%) (*p* = 0.005). The overall incidence of regraft rejection was 10.6%; regraft rejection was also more common among PK (23.8%) compared to EK (6.9%) and DALK regrafts (5.3%) (*p* = 0.001). The single case of rejection in a DALK regraft resolved with medical treatment. There were only 5 cases of microbial keratitis (1.8%), which occurred in all three groups (DALK 5.3%, PK 3.2%, EK 1.0%, *p* = 0.108).

### 3.3 Regraft success and cumulative survival probabilities

The overall regraft success rate at one year was 88.4%. No significant differences in success rates were found between PK/PK and PK/EK (75.9% vs. 89.2% respectively, *p* = 0.053), and EK/PK and EK/EK (80.0% vs. 91.7% respectively, *p* = 0.372; Supplementary Figure S1). EK/EK had a significantly higher success rate than PK/PK (91.7% vs. 75.9%, *p* = 0.004), while no difference was seen between PK/EK and EK/EK (89.2% vs. 91.7%, *p* = 0.577). Regraft success rates for initial DALK grafts were 100% regardless of regraft type while that of EK/DALK was 88.9%.

**Figure 1:**
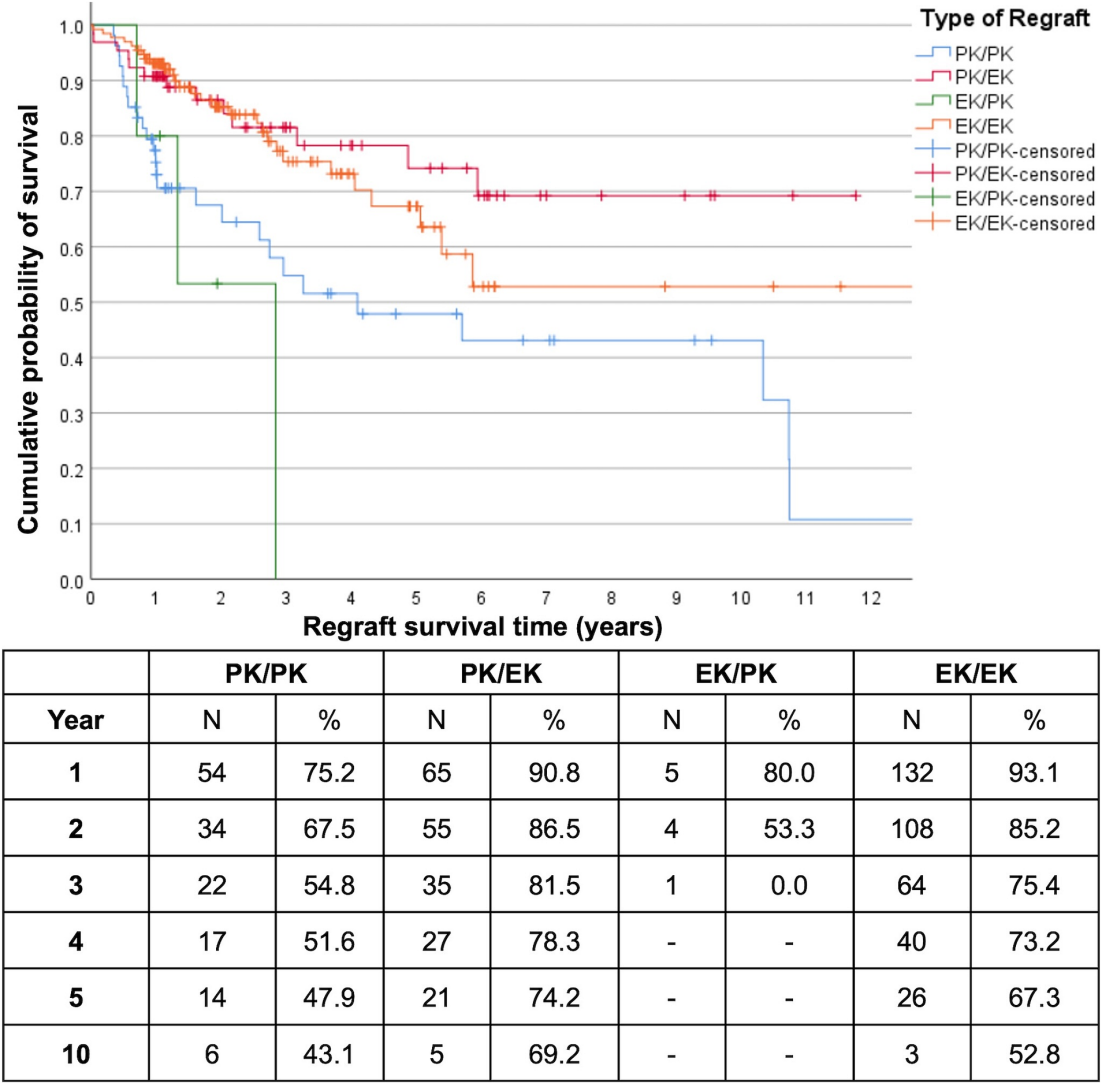
Kaplan–Meier survival plot of first repeat optical keratoplasties according to type of graft/regraft (*n* = 256 (59 PK, 197 EK)). Censored data indicate last follow-up for each patient. PK = penetrating keratoplasty; EK = endothelial keratoplasty.

**Figure 2:**
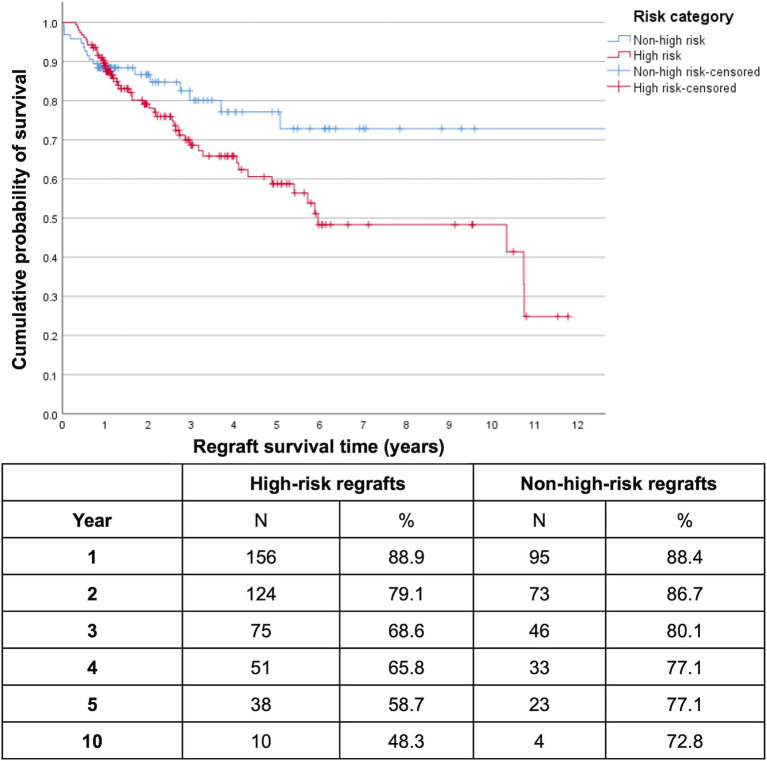
Kaplan–Meier survival plot of high-risk versus non-high-risk first repeat optical keratoplasties (*n* = 251 (54 PK, 197 EK)). Censored data indicate last follow-up for each patient. High-risk regrafts had significantly lower 10-year survival (48.3%) compared to non-high-risk regrafts (72.8%) (log-rank *p* = 0.045).

At the end of the first and second postoperative years, overall cumulative regraft survival probabilities were similar for PK/EK (90.8 and 86.5% respectively) and EK/EK (93.1 and 85.2% respectively) while PK/PK had already decreased to 75.2% by the first year (Figure 1). Five- and 10-year regraft survival probabilities were 74.2 and 69.2% for PK/EK, 67.3 and 52.8% for EK/EK, and 47.9 and 43.1% for PK/PK. There were only 5 EK/PK grafts, none of which were seen to survive past year 3. Log-rank test showed greater regraft survival probabilities of PK/EK compared to PK/PK (*p* = 0.002) and to EK/PK (*p* = 0.009), and of EK/EK compared to PK/PK (*p* = 0.003) and to EK/PK (*p* = 0.005). No difference in graft survival probabilities were found between PK/EK and EK/EK (*p* = 0.434), and between PK/PK and EK/PK (*p* = 0.390). DALK/PK and DALK/DALK regrafts maintained 100% survival for at least 5 years, while DALK/EK exhibited 100% survival for at least 3 years. High-risk cases were found to have significantly lower 10-year regraft survival (48.3%) compared to non-high-risk cases (72.8%) (*p* = 0.045; Figure 2).

There were 73 first regrafts (25.7%) that failed during the study period. Table 3 shows the reasons for failure, with some grafts having more than one cause. The most common was late endothelial failure (50.7% of failed regrafts), followed by graft rejection (26.0%) and increased IOP (19.2%). Rates of late endothelial failure were similar between PK regrafts and EK regrafts (*p* = 0.157), while regraft rejection was more frequently seen among PK regrafts (*p* = 0.024). Increased IOP as a reason for failure was similar among PK (20.7%) and EK regrafts (19.5%). Overall, late endothelial failure and regraft rejection were the top causes of failure for PK regrafts (41.4% each) while for EK regrafts, late endothelial failure (58.5%) was more common than regraft rejection (17.1%). Three DALK regrafts (DSAEK/DALK and DALK/DALK) failed in our study, due to endothelial failure of the initial DSAEK graft, infection, or recurrence of primary disease.

**Table 3:** Causes of failure of the 73 failed first regrafts.

Cause of regraft failure	All failed regrafts *n* = 73 (25.7% of all regrafts)	Failed PK as regraft *n* = 29 (46.0% of PK regrafts)	Failed EK as regraft *n* = 41 (20.3% of EK regrafts)	Failed DALK as regraft *n* = 3 (15.8% of DALK regrafts)
Late endothelial failure	37 (50.7%)	12 (41.4%)	24 (58.5%)	1 (33.3%)
Regraft rejection	19 (26.0%)	12 (41.4%)	7 (17.1%)	0 (0.0%)
Increased IOP	14 (19.2%)	6 (20.7%)	8 (19.5%)	0 (0.0%)
Epitheliopathy	6 (8.2%)	3 (10.3%)	3 (7.3%)	0 (0.0%)
Microbial keratitis	4 (5.5%)	2 (6.9%)	1 (2.4%)	1 (33.3%)
Recurrence of primary disease	4 (5.5%)	1 (3.4%)	2 (4.9%)	1 (33.3%)
Primary graft failure	2 (2.7%)	1 (3.4%)	1 (2.4%)	0 (0.0%)
Others^a^	5 (6.8%)	1 (3.4%)	4 (9.8%)	0 (0.0%)
Total causes of graft failure	91 (100%)	38 (100%)	50 (100%)	3 (100%)

PK, penetrating keratoplasty; EK, endothelial keratoplasty; DALK, deep anterior lamellar keratoplasty. ^a^EK regrafts: 3 cases failed due to Cytomegalovirus endotheliitis, 1 case due to phthisis bulbi. PK regrafts: 1 case failed due to endotheliitis. The values in the table represent the numbers of eyes and their percentage among the failed grafts. The total number of causes exceeds the total number of eyes since some grafts had more than one reason for failure.

### 3.4 Postoperative BCVA

Preoperative and one-year postoperative logMAR BCVA of 84 patients according to type of first graft and regraft are summarized in Table 4. To account for confounders, analysis excluded cases with limited visual potential not due to corneal factors, while still including failed grafts. Overall median preoperative logMAR BCVA was 1.33 ± 0.47 while postoperative logMAR BCVA at 1 year was 0.55 ± 0.50. There was a significant difference in the baseline BCVA of failed initial PK and initial EK grafts (PK/PK, EK/PK, PK/EK, and EK/EK) (*p* = 0.027). Those that eventually underwent PK regrafting generally had worse median baseline VA than those that underwent EK regrafting. After 1 year, the worst visual outcomes were seen in PK/PK cases (*p* = 0.001). PK/PK was also found to have worse preoperative and postoperative BCVA compared to PK/EK (pre *p* = 0.014, post *p* = 0.006), and EK/EK (pre *p* = 0.005, post *p* < 0.001). PK/EK and EK/EK both had similar preoperative and good postoperative visual outcomes (pre *p* = 0.893, post *p* = 0.295).

**Table 4:** Best corrected visual acuities in logarithm of the minimum angle of resolution at 1 year according to type of graft/regraft (*n* = 80).

Graft/regraft	Number of grafts (excluding eyes with co-morbidities) (% of total grafts)	Mean preoperative logMAR BCVA (SD)	Mean one-year postoperative logMAR BCVA (SD)
PK/PK	10 (18.5)	1.73 (0.37)	1.07 (0.66)
PK/EK	16 (24.6)	1.36 (0.43)	0.53 (0.49)
EK/EK	44 (33.3)	1.36 (0.43)	0.43 (0.37)
EK/PK	1 (20.0)	1.7	0.2
DALK/PK	1 (25.0)	0.9	0.2
DALK/DALK	7 (70.0)	0.73 (0.44)	0.63 (0.63)
DALK/EK	1 (20.0)	1.3	0.5
EK/DALK	4 (44.4)	1.03 (0.19)	0.78 (0.64)
All grafts	84 (29.6)	1.33 (0.47)	0.55 (0.50)

logMAR, logarithm of the minimum angle of resolution; BCVA, best corrected visual acuity; SD, standard deviation; PK, penetrating keratoplasty; EK, endothelial keratoplasty; DALK, deep anterior lamellar keratoplasty; SD, standard deviation. Failed regrafts were included, while cases with other comorbidities potentially limiting visual potential (cataract, amblyopia, increased IOP, optic atrophy, and vitreoretinal disease) in addition to those without recorded postoperative best corrected visual acuity data were excluded.

Due to the small number of DALK grafts with postoperative BCVA, no analysis was done although the DALK/DALK group had better preoperative BCVA and a smaller absolute improvement in BCVA compared to solitary cases of DALK/PK and DALK/EK.

### 3.5 Cox regression analysis

A Cox proportional hazards regression model was constructed to assess risk factors associated with regraft failure. We found male gender (hazard ratio (HR) 1.943, *p* = 0.023), PK regraft (HR 2.267, *p* = 0.003), and regraft rejection (HR 2.945, p = 0.003) to be significant risk factors for failure of first regrafts (Table 5). Longer time between first and second grafts on the other hand was associated with a decreased risk of regraft failure (HR 0.909, *p* = 0.013). Looking at the indications for the initial graft, PBK (HR 5.764, *p* = 0.005), ABK (HR 8.124, *p* = 0.004), and other causes of scarring or edema (HR 10.925, *p* < 0.001) were associated with a higher risk for regraft failure compared to FED. Although univariable analysis suggested decreased graft survival in patients having preoperative increased IOP (Supplementary Table S5), multivariable analysis did not show this to be significant. Age, race, regraft indication, and having combined intraocular surgery did not affect graft survival in our study.

**Table 5:** Multivariate cox regression analysis of factors found to be significant in univariate analysis for regraft failure (*n* = 284).

Predictor	*n*	Hazard ratio	*p* > |z|	95% CI	
				Lower	Upper
Gender					
Male	164	1.943	0.023	1.096	3.443
Female	120	ref = 1			
Regraft procedure					
PK	63	2.267	0.003	1.321	3.893
DALK	19	0.716	0.603	0.204	2.515
EK	202	ref = 1	0.006		
Increased IOP					
Yes	144	1.250	0.407	0.737	2.122
No	140	ref = 1			
First graft indication					
Fuchs endothelial dystrophy	50	ref = 1	0.007		
Pseudophakic bullous keratopathy	108	5.764	0.005	1.689	19.675
Other causes of scarring/edema	40	10.925	<0.001	2.872	41.565
Corneal dystrophy aside from FED and keratoconus	22	2.472	0.227	0.569	10.728
Keratoconus	17	1.690	0.579	0.265	0.779
Aphakic bullous keratopathy	16	8.124	0.004	1.920	34.378
Post infectious scar/ thinning	14	3.725	0.119	0.712	19.501
Corneal injury	14	3.422	0.131	0.693	16.906
Others	3	17.386	0.003	2.730	110.712
Time between grafts (years)	284	0.909	0.013	0.843	0.980
Regraft rejection					
Yes	20	2.945	0.003	1.455	5.960
No	264	ref = 1			

CI, confidence interval; ref, reference; PK, penetrating keratoplasty; DALK, deep anterior lamellar keratoplasty; EK, endothelial keratoplasty; IOP, intraocular pressure; FED, Fuchs endothelial dystrophy.

## 4 Discussion

To our knowledge, this study is the first to report 10-year first regraft survival probabilities, which were significantly higher in PK/EK and in EK/EK compared to PK/PK regrafts. We found postoperative BCVA at 1 year among PK/EK and EK/EK regrafts to be better compared to repeat PK. Postoperative complications of regraft rejection as well as increased IOP were more frequently seen among PK regrafts compared to EK regrafts. Rejection and failure rates for DALK/DALK regrafts in our study were low, and 100% 3- to 5-year survival probabilities were seen for all types of regrafts performed for failed optical DALK. Regrafts which had one or more additional preoperative risk factors for failure demonstrated significantly lower 10-year survival than those which did not. Through multivariate analyses, male gender, initial graft indications of PBK and ABK, regraft rejection, and having a PK regraft versus an EK regraft were found to be significant risk factors for regraft failure while longer time between first and second grafts was associated with a lower risk of failure.

Five-year regraft survival probabilities in our study mostly concurred with those reported in similar published studies (42.6–65.6% for PK/PK, 38.8–86.4% for PK/EK, 50.1–81% for EK/EK) (7, 9, 12). Variations in these estimates are attributed to various center- and surgeon-related factors, as well characteristics inherent in each study cohort. For example, endothelial failure among Asian eyes is most often due to PBK as in this study, unlike in Caucasian eyes wherein FED is more common (7, 21, 22).

Studies have shown EK to have longer graft survival and lower risk of immunologic rejection compared to PK (23), possibly due to the smaller amount of tissue transplanted as well as the absence of sutures which could incite a rejection episode (5, 6, 10, 24–26). However, not all studies have been able to find a clear benefit of EK compared to PK regraft in terms of graft survival (6–9, 12, 13). It should be noted that some of these studies were large, long-term registry studies involving multiple centers and more corneal surgeons with variations in surgical experience and techniques as well as in postoperative regimens (6, 9, 12). Surgeons could also have still been in the DSAEK learning curve as some studies included regrafts performed in the early 2000s (9, 12). Keane et al. found higher rates of graft detachment and primary graft failure of EK under PK which may reflect a learning curve among less experienced surgeons (6). Unlike in multicenter registry studies, all surgeons in our single-center study use standardized techniques and postoperative treatment regimens with relatively equal follow-up times. Another strength of our study is its limitation to the first repeat grafts to avoid the confounding effect of progressively decreasing graft survival with increasing number of previous grafts (6). This is unlike many other studies which included all repeat grafts regardless of sequence (3, 5, 8, 10, 24, 27, 28). Our overall 5-year survival probability for DALK/DALK was higher than those reported by two other similar single-center studies (38.8% (29) and 78.3% (15)). Those studies however included cases from the 1990s, and our results could reflect subsequent improvements in stromal dissection methods especially the big-bubble technique introduced in 2002 (30).

Graft rejection and late endothelial failure have been cited as the most frequent reasons for graft failure requiring repeat corneal transplantations (3, 7, 9, 12, 28). Failure of the first graft due to rejection was seen in 38.4% of regrafts in our study, with estimates in the literature ranging from 17 to 48.3% (3, 6, 7, 9, 12). A rejection episode in any graft or regraft is associated with a significantly greater risk of failure for that graft, in addition to further episodes of rejection (5, 6, 28). Prior graft failure is inherently another high-risk factor in repeat keratoplasty, especially if it was due to allograft rejection (3, 27, 31). Repeat transplant patients at our center undergo more frequent follow-ups and slower tapering of topical corticosteroids. High-risk patients are given additional topical and systemic immunosuppression. This requires collaboration with a rheumatologist and close monitoring of blood chemistry.

In our study, only 15.8% of the primary DALK grafts had failed due to stromal rejection while only one DALK regraft experienced a rejection episode, which subsequently resolved with topical prednisolone. This could explain the high survival seen among all types of regrafts performed after a failed DALK. Our group’s previous paper also reported similarly low rates of stromal rejection in repeat ALK compared to primary DALK (15).

We also found that a longer time to regraft was associated with decreased risk of regraft failure. Claesson et al. and Keane et al. found improved regraft survival especially if the initial graft survived at least 2–5 years (6, 10). In our study, preoperative increased IOP was a significant risk factor for regraft failure in univariate analysis but was not retained in multivariate analysis. This could be due to its correlation with another risk factor such as regraft rejection. Lu et al. in their multi-center study reported that having a concurrent surgical procedure during transplantation was associated with increased regraft failure, although this was not significant in our study (28).

In the original Singapore Corneal Transplant Study, male sex was also found to be a significant risk factor for failure of primary PK grafts (32). One recent study found a significantly higher risk of repeat keratoplasty for males compared to females (33), while another did not observe gender to affect regraft survival or rejection (27). The reasons for this are not yet well understood. Shin et al. proposed that male patients could have more resources or support for having repeat surgery or have an etiology with less favorable outcomes than females (33). Another factor to consider is donor-recipient sex compatibility, although the evidence for sex-matching is still not conclusive (34–36).

In contrast to some published studies, this study showed significantly better one-year postoperative BCVA in PK/EK grafts compared to PK/PK, and in EK/EK grafts when compared to PK/PK grafts, although mean preoperative BCVA between the regraft groups also differed significantly (6, 12, 13). Kitzmann et al. found similar median BCVA in PK/PK and PK/DSAEK after 1 year, although significantly better final visual acuity was seen in the latter after excluding failed grafts (8).

The authors recognize the limitations to this study such as its modest sample size and retrospective design. There were much fewer DMEK than DSAEK regrafts since the DMEK technique was introduced more recently and may also be less preferred over DSAEK as a repeat graft. The learning curve is already significant with performing DMEK as a primary technique, and it is even more difficult to do in complex eyes with severely edematous failed grafts and alteration of anterior segment architecture (17). Due to the low number of DMEK regrafts, comparison with DSAEK was not possible in the present study. Analysis will be performed once we have more long-term data on regrafts performed using DMEK.

Another limitation of the study is the longer median follow-up time of PK regrafts compared to DSAEK and especially DMEK regrafts, thereby potentially underestimating the long-term complications or regraft failure rates for EK. This could be partially offset by having a greater number of EK than PK regrafts in our population, unlike in other studies (3, 6–9, 12, 13, 28). The numbers of previous rejection episodes could also be underreported as mild episodes may subsequently resolve with adherence to the current immunosuppressive regimen without consulting in clinic. Despite these limitations, our graft registry data remains valuable as it represents real-world data in an Asian population. In addition, not all patients in our study reached 5 years of follow-up; median follow-up was 2 years but Kaplan–Meier analysis adjusts for this by censoring data from lost cases.

## 5 Conclusion

In conclusion, our study showed that performing EK for a failed optical PK or EK significantly improved regraft survival compared to repeat PK. Repeat EK was also associated with higher regraft survival and success rates compared to repeat PK. Regrafts performed for failed initial DALK grafts did well regardless of type.

## Data Availability

The original contributions presented in the study are included in the article/Supplementary material. Further inquiries regrding additional data can be directed to the corresponding author upon reasonable request.

## References

[1] Alio Del Barrio JL Bhogal M Ang M Ziaei M Robbie S Montesel A. Corneal transplantation after failed grafts: options and outcomes. Surv Ophthalmol. (2021) 66:20–40. doi: 10.1016/j.survophthal.2020.10.003, PMID: 33065176

[2] Crawford AZ Patel DV McGhee C. A brief history of corneal transplantation: from ancient to modern. Oman J Ophthalmol. (2013) 6:S12–7. doi: 10.4103/0974-620X.122289, PMID: 24391366 PMC3872837

[3] Wan X Yao W Zhao S Xu J Le Q. Indications and surgical techniques for repeat corneal transplantation in eastern China: a twelve-year study. J Ophthalmol. (2021) 2021:1–8. doi: 10.1155/2021/5514004, PMID: 34631163 PMC8497152

[4] Wang F Zhang T Kang YW He JL Li SM Li SW. Endothelial keratoplasty versus repeat penetrating keratoplasty after failed penetrating keratoplasty: a systematic review and meta-analysis. PLoS One. (2017) 12:e0180468. doi: 10.1371/journal.pone.0180468, PMID: 28671976 PMC5495398

[5] Einan-Lifshitz A Mednick Z Belkin A Sorkin N Alshaker S Boutin T. Comparison of Descemet stripping automated endothelial Keratoplasty and Descemet membrane endothelial Keratoplasty in the treatment of failed penetrating Keratoplasty. Cornea. (2019) 38:1077–82. doi: 10.1097/ICO.0000000000001993, PMID: 31394551

[6] Keane MC Galettis RA Mills RA Coster DJ. Williams KA, for contributors to the Australian corneal graft R. A comparison of endothelial and penetrating keratoplasty outcomes following failed penetrating keratoplasty: a registry study. Br J Ophthalmol. (2016) 100:1569–75. doi: 10.1136/bjophthalmol-2015-307792, PMID: 26892633

[7] Ang M Ho H Wong C Htoon HM Mehta JS Tan D. Endothelial keratoplasty after failed penetrating keratoplasty: an alternative to repeat penetrating keratoplasty. Am J Ophthalmol. (2014) 158:1221–1227.e1. doi: 10.1016/j.ajo.2014.08.024, PMID: 25152499

[8] Kitzmann AS Wandling GR Sutphin JE Goins KM Wagoner MD. Comparison of outcomes of penetrating keratoplasty versus Descemet's stripping automated endothelial keratoplasty for penetrating keratoplasty graft failure due to corneal edema. Int Ophthalmol. (2012) 32:15–23. doi: 10.1007/s10792-012-9518-4, PMID: 22271071

[9] Aboshiha J Jones MNA Hopkinson CL Larkin DFP. Differential survival of penetrating and lamellar transplants in Management of Failed Corneal Grafts. JAMA Ophthalmol. (2018) 136:859–65. doi: 10.1001/jamaophthalmol.2018.1515, PMID: 29931227 PMC6142952

[10] Claesson M Armitage WJ. Clinical outcome of repeat penetrating keratoplasty. Cornea. (2013) 32:1026–30. doi: 10.1097/ICO.0b013e31828a2810, PMID: 23591148

[11] Feizi S Javadi MA Khajuee-Kermani P Jafari R. Repeat Keratoplasty for failed deep anterior lamellar Keratoplasty in keratoconus: incidence, indications, and outcomes. Cornea. (2017) 36:535–40. doi: 10.1097/ICO.0000000000001169, PMID: 28257387

[12] Dickman MM Spekreijse LS Dunker SL Winkens B Berendschot T van den Biggelaar F. Long-term outcomes of repeated corneal transplantations: a prospective Dutch registry study. Am J Ophthalmol. (2018) 193:156–65. doi: 10.1016/j.ajo.2018.06.018, PMID: 29963996

[13] Ramamurthy S Reddy JC Vaddavalli PK Ali MH Garg P. Outcomes of repeat Keratoplasty for failed therapeutic Keratoplasty. Am J Ophthalmol. (2016) 162:e2:83–88.e2. doi: 10.1016/j.ajo.2015.11.004, PMID: 26558523

[14] Faramarzi A Abbasi H Feizi S Hadi Y Azari AA Karimian F. Topical 0.03% tacrolimus versus systemic mycophenolate mofetil as adjuncts to systemic corticosteroids for preventing graft rejection after repeat keratoplasty: one-year results of a randomized clinical trial. Eye (Lond). (2021) 35:2879–88. doi: 10.1038/s41433-020-01375-z, PMID: 33414533 PMC8452649

[15] Woo JH Tan YL Htoon HM Tan DTH Mehta JS. Outcomes of repeat anterior lamellar Keratoplasty. Cornea. (2020) 39:200–6. doi: 10.1097/ICO.0000000000002167, PMID: 31584477

[16] Bohringer D Grotejohann B Ihorst G Reinshagen H Spierings E Reinhard T. Rejection prophylaxis in corneal transplant. Dtsch Arztebl Int. (2018) 115:259–65. doi: 10.3238/arztebl.2018.0259, PMID: 29735006 PMC5949374

[17] Woo JH Ang M Htoon HM Tan D. Descemet membrane endothelial Keratoplasty versus Descemet stripping automated endothelial Keratoplasty and penetrating Keratoplasty. Am J Ophthalmol. (2019) 207:288–303. doi: 10.1016/j.ajo.2019.06.012, PMID: 31228467

[18] Ong HS Htoon HM Ang M Mehta JS. "endothelium-out" and "endothelium-in" Descemet membrane endothelial Keratoplasty (DMEK) graft insertion techniques: a systematic review with Meta-analysis. Front Med (Lausanne). (2022) 9:868533. doi: 10.3389/fmed.2022.868533, PMID: 35775001 PMC9237218

[19] Tan DT Mehta JS. Future directions in lamellar corneal transplantation. Cornea. (2007) 26:S21–8. doi: 10.1097/ICO.0b013e31812f685c, PMID: 17881911

[20] Huang OS Mehta JS Htoon HM Tan DT Wong TT. Incidence and risk factors of elevated intraocular pressure following deep anterior lamellar Keratoplasty. Am J Ophthalmol. (2016) 170:153–60. doi: 10.1016/j.ajo.2016.07.025, PMID: 27519560

[21] Anshu A Li L Htoon HM de Benito-Llopis L Shuang LS Singh MJ. Long-term review of penetrating Keratoplasty: a 20-year review in Asian eyes. Am J Ophthalmol. (2021) 224:254–66. doi: 10.1016/j.ajo.2020.10.014, PMID: 33129808

[22] Writing Committee for the Cornea Donor Study Research Mannis MJ Holland EJ Gal RL Dontchev M Kollman C. The effect of donor age on penetrating keratoplasty for endothelial disease: graft survival after 10 years in the cornea donor study. Ophthalmology. (2013) 120:2419–27. doi: 10.1016/j.ophtha.2013.08.026 24246825 PMC3885822

[23] Ang M He F Lang S Sabanayagam C Cheng CY Arundhati A. Machine learning to analyze factors associated with ten-year graft survival of Keratoplasty for cornea endothelial disease. Front Med (Lausanne). (2022) 9:831352. doi: 10.3389/fmed.2022.831352, PMID: 35721073 PMC9200960

[24] Zafar S Wang P Woreta FA Aziz K Makary M Srikumaran D. Risk factors for repeat Keratoplasty after endothelial Keratoplasty in the Medicare population. Am J Ophthalmol. (2021) 221:287–98. doi: 10.1016/j.ajo.2020.08.006, PMID: 32791066

[25] Ang M Soh Y Htoon HM Mehta JS Tan D. Five-year graft survival comparing Descemet stripping automated endothelial Keratoplasty and penetrating Keratoplasty. Ophthalmology. (2016) 123:1646–52. doi: 10.1016/j.ophtha.2016.04.049, PMID: 27262764

[26] Ong HS Ang M Mehta J. Evolution of therapies for the corneal endothelium: past, present and future approaches. Br J Ophthalmol. (2021) 105:454–67. doi: 10.1136/bjophthalmol-2020-316149, PMID: 32709756 PMC8005807

[27] Mitry D Bhogal M Patel AK Lee BS Chai SM Price MO. Descemet stripping automated endothelial keratoplasty after failed penetrating keratoplasty: survival, rejection risk, and visual outcome. JAMA Ophthalmol. (2014) 132:742–9. doi: 10.1001/jamaophthalmol.2014.352, PMID: 24763830

[28] Lu LM Boyle AB Niederer RL Brookes NH McGhee CNJ Patel DV. Repeat corneal transplantation in Auckland, New Zealand: indications, visual outcomes and risk factors for repeat keratoplasty failure. Clin Experiment Ophthalmol. (2019) 47:987–94. doi: 10.1111/ceo.13581, PMID: 31268240

[29] Yasu-Mimura R Hirayama M Kasamatsu H Yamaguchi T Shimazaki J. Etiology-specific comparison of the long-term clinical outcome of repeat deep anterior lamellar Keratoplasty for optical indications. Cornea. (2023) 42:598–606. doi: 10.1097/ICO.0000000000003189, PMID: 36727893

[30] Anwar M Teichmann KD. Big-bubble technique to bare Descemet's membrane in anterior lamellar keratoplasty. J Cataract Refract Surg. (2002) 28:398–403. doi: 10.1016/S0886-3350(01)01181-6, PMID: 11973083

[31] Jabbehdari S Rafii AB Yazdanpanah G Hamrah P Holland EJ Djalilian AR. Update on the Management of High-Risk Penetrating Keratoplasty. Curr Ophthalmol Rep. (2017) 5:38–48. doi: 10.1007/s40135-017-0119-2, PMID: 28959505 PMC5612422

[32] Tan DT Janardhanan P Zhou H Chan YH Htoon HM Ang LP. Penetrating keratoplasty in Asian eyes: the Singapore corneal transplant study. Ophthalmology. (2008) 115:975–982.e1. doi: 10.1016/j.ophtha.2007.08.049, PMID: 18061267

[33] Shin KY Lim DH Han K Chung TY. Higher incidence of penetrating keratoplasty having effects on repeated keratoplasty in South Korea: a nationwide population-based study. PLoS One. (2020) 15:e0235233. doi: 10.1371/journal.pone.0235233, PMID: 32628736 PMC7337353

[34] Volker-Dieben HJ Kok-van Alphen CC Lansbergen Q Persijn GG. Different influences on corneal graft survival in 539 transplants. Acta Ophthalmol. (1982) 60:190–202. doi: 10.1111/j.1755-3768.1982.tb08373.x, PMID: 6753453

[35] Ong HS Chiam N Htoon HM Kumar A Arundhati A Mehta JS. The effects of donor-recipient age and sex compatibility in the outcomes of deep anterior lamellar Keratoplasties. Front Med (Lausanne). (2021) 8:801472. doi: 10.3389/fmed.2021.801472, PMID: 35155480 PMC8828935

[36] Price DA Kelley M Price FW Jr Price MO. Five-year graft survival of Descemet membrane endothelial Keratoplasty (EK) versus Descemet stripping EK and the effect of donor sex matching. Ophthalmology. (2018) 125:1508–14. doi: 10.1016/j.ophtha.2018.03.050, PMID: 29731147

