# Supplementary material for: Ten-year outcomes of repeat keratoplasty for optical indications
Source: Front Med (Lausanne). 2025 Jan 22;11:1503333. doi: 10.3389/fmed.2024.1503333 (PMC11796611; doi:10.3389/fmed.2024.1503333)
Supplement: Supplementary file 1 [file Table_1.docx]

**Supplementary Table 1.** General criteria used by the Singapore Eye Bank for optical donor tissue.

|  | **PK** | **DSAEK/DMEK** | **DALK** |
| --- | --- | --- | --- |
| **Death- to- surgery interval** | <7 days | <7 days | Beyond 7 days acceptable up to 14 days |
| **Donor age (years)** | 2 to 80 | DSAEK: 20 and above  DMEK: 50 and above | 2 to 80 |
| **Central clear stromal zone** | >8 mm | N/A | >8 mm |
| **Endothelial cell density** | > 2,250 cells/mm^2^ | > 2,500 cells/mm^2^ | <2,250 cells/mm^2^ acceptable |
| **Other criteria** | No pathology in stroma (e.g. scars) | DSAEK: uniform rim, at least 18 mm in diameter, no prior refractive surgery, no pathology in Descemet membrane  (e.g. tears) or endothelium  DMEK: no diabetes, no jaundice, no pathology in Descemet membrane (e.g. tears) or endothelium | No pathology in stroma (e.g. scars) |

PK, penetrating keratoplasty; DSAEK, Descemet stripping automated endothelial keratoplasty; DMEK, Descemet membrane endothelial keratoplasty; DALK, deep anterior lamellar keratoplasty
