# Supplementary material for: Ten-year outcomes of repeat keratoplasty for optical indications
Source: Front Med (Lausanne). 2025 Jan 22;11:1503333. doi: 10.3389/fmed.2024.1503333 (PMC11796611; doi:10.3389/fmed.2024.1503333)
Supplement: Supplementary file 2 [file Table_2.docx]

**Supplementary Table 2.** Length of time in years from primary graft to first regraft by graft type (n=284).

| Time (yrs) | **All regrafts** | **PK/ PK**  **(n=54)** | **PK/ DSAEK**  **(n=63)** | **PK/ DMEK**  **(n=2)** | **DALK/ PK**  **(n=4)** | **DALK/ DALK**  **(n=10)** | **DALK/ DSAEK**  **(n=4)** | **DALK/ DMEK**  **(n=1)** | **DSAEK/ PK**  **(n=4)** | **DSAEK/ DALK**  **(n=9)** | **DSAEK/ DSAEK**  **(n=106)** | **DSAEK/ DMEK**  **(n=13)** | **DMEK/ PK**  **(n=1)** | **DMEK/ DSAEK**  **(n=8)** | **DMEK/ DMEK**  **(n=5)** |
| --- | --- | --- | --- | --- | --- | --- | --- | --- | --- | --- | --- | --- | --- | --- | --- |
| Mean ± SD | 5.2 ± 4.7 | 6.5 ± 4.9 | 9.1 ± 5.7 | 9.7 ± 4.3 | 2.0 ± 2.8 | 2.2 ± 1.7 | 2.3 ± 2.2 | 2.3 | 2.8 ± 4.5 | 2.4 ± 1.4 | 3.3 ± 2.3 | 6.3 ± 2.5 | 0.3 | 0.4 ± 0.4 | 0.6 ± 0.6 |
| Median | 3.9 | 5.6 | 8.0 | 9.7 | 0.9 | 2.1 | 2.0 | 2.3 | 0.8 | 1.8 | 3.1 | 6.9 | 0.3 | 0.3 | 0.6 |
| Range | 4 days – 26 yrs | 8 days – 22 yrs | 1 – 26 yrs | 7 – 13 yrs | 45 days – 6 yrs | 42 days – 6 yrs | 0.2 – 5 yrs | - | 14 days – 10 yrs | 1 – 5 yrs | 5 days – 10 yrs | 3 – 10 yrs | - | 4 days – 1 yr | 13 days – 2 yrs |

PK, penetrating keratoplasty; DSAEK, Descemet stripping automated endothelial keratoplasty; DMEK, Descemet membrane endothelial keratoplasty; DALK, deep anterior lamellar keratoplasty; SD, standard deviation; yr(s), year(s)
