# Supplementary material for: Ten-year outcomes of repeat keratoplasty for optical indications
Source: Front Med (Lausanne). 2025 Jan 22;11:1503333. doi: 10.3389/fmed.2024.1503333 (PMC11796611; doi:10.3389/fmed.2024.1503333)
Supplement: Supplementary file 3 [file Table_3.docx]

**Supplementary Table 3.** Types of primary grafts and respective regrafts performed (n=284).

|  | **Regraft Type** | | | | **Total first grafts (%)** |
| --- | --- | --- | --- | --- | --- |
| **First graft type** | **PK (%)** | **DSAEK (%)** | **DMEK (%)** | **DALK (%)** |  |
| **PK** | 54 (19.0) | 63 (22.2) | 2 (0.7) | 0 (0.0) | 119 (41.9) |
| **DSAEK** | 4 (1.4) | 106 (37.3) | 13 (4.6) | 9 (3.2) | 132 (46.5) |
| **DMEK** | 1 (0.4) | 8 (2.8) | 5 (1.8) | 0 (0.0) | 14 (4.9) |
| **DALK** | 4 (1.4) | 4 (1.4) | 1 (0.4) | 10 (3.5) | 19 (6.7) |
| **Total regrafts** | 63 (22.2) | 181 (63.7) | 21 (7.4) | 19 (6.7) | 284 (100) |

PK, penetrating keratoplasty; DSAEK, Descemet stripping automated endothelial keratoplasty; DMEK, Descemet membrane endothelial keratoplasty; DALK, deep anterior lamellar keratoplasty
