# Supplementary material for: Ten-year outcomes of repeat keratoplasty for optical indications
Source: Front Med (Lausanne). 2025 Jan 22;11:1503333. doi: 10.3389/fmed.2024.1503333 (PMC11796611; doi:10.3389/fmed.2024.1503333)
Supplement: Supplementary file 4 [file Table_4.docx]

**Supplementary Table 4.** Intraocular procedures combined with regrafting (n=47). Some cases had more than one additional procedure, each of which was counted under its respective category.

| **Type of regraft** | **Phaco** | **IOL suturing/ repositioning/ exchange/ explantation** | **Anterior vitrectomy** | **Silicone oil removal** | **Graft refractive surgery** |
| --- | --- | --- | --- | --- | --- |
| **PK/PK** | 7 | 2 | 5 | 1 | 0 |
| **PK/EK** | 5 | 2 | 2 | 0 | 1 |
| **DALK/PK** | 3 | 0 | 0 | 0 | 0 |
| **DALK/EK** | 4 | 0 | 0 | 0 | 0 |
| **EK/EK** | 4 | 9 | 5 | 0 | 0 |
| **Total procedures** | 23 | 13 | 12 | 1 | 1 |

Phaco, phacoemulsification; IOL, intraocular lens; PK, penetrating keratoplasty; EK, endothelial keratoplasty; DALK, deep anterior lamellar keratoplasty
