# Supplementary material for: Ten-year outcomes of repeat keratoplasty for optical indications
Source: Front Med (Lausanne). 2025 Jan 22;11:1503333. doi: 10.3389/fmed.2024.1503333 (PMC11796611; doi:10.3389/fmed.2024.1503333)
Supplement: Supplementary file 5 [file Table_5.docx]

**Supplementary Table 5.** Univariate Cox regression analysis comparing baseline characteristics and regraft failure (n=284).

| **Predictor** | **n** | **Hazard ratio** | **P>\|z\|** | **95% CI** | |
| --- | --- | --- | --- | --- | --- |
|  |  |  |  | Lower | Upper |
| **Age** | 284 | 1.009 | 0.335 | 0.991 | 1.026 |
| **Gender** |  |  |  |  |  |
| Male | 164 | 2.291 | 0.002 | 1.369 | 3.835 |
| Female | 120 | ref=1 |  |  |  |
| **Race** |  |  |  |  |  |
| Chinese | 217 | ref=1 | 0.559 |  |  |
| Malay | 28 | 0.721 | 0.448 | 0.310 | 1.676 |
| Indian | 20 | 0.623 | 0.361 | 0.226 | 1.720 |
| Others | 19 | 0.563 | 0.334 | 0.176 | 1.803 |
| **Regraft procedure** |  |  |  |  |  |
| PK | 63 | 2.351 | <0.001 | 1.456 | 3.796 |
| DALK | 19 | 0.689 | 0.535 | 0.213 | 2.229 |
| EK | 202 | ref=1 | 0.001 |  |  |
| **Preoperative glaucoma/ increased IOP** |  |  |  |  |  |
| Yes | 144 | 2.123 | 0.003 | 1.302 | 3.464 |
| No | 140 | ref=1 |  |  |  |
| **First graft indication** |  |  |  |  |  |
| Fuchs endothelial dystrophy | 50 | ref=1 | 0.004 |  |  |
| Pseudophakic bullous keratopathy | 108 | 8.816 | <0.001 | 2.693 | 28.862 |
| Other causes of scarring/edema | 40 | 10.488 | <0.001 | 2.917 | 37.714 |
| Corneal dystrophy aside from FED and keratoconus | 22 | 3.604 | 0.080 | 0.858 | 15.123 |
| Keratoconus | 17 | 2.288 | 0.365 | 0.381 | 13.731 |
| Aphakic bullous keratopathy | 16 | 9.127 | 0.001 | 2.354 | 35.391 |
| Post infectious scar/ thinning | 14 | 4.309 | 0.074 | 0.867 | 21.417 |
| Corneal injury | 14 | 4.719 | 0.043 | 1.053 | 21.153 |
| Others | 3 | 19.847 | 0.001 | 3.276 | 120.256 |
| **Regraft indication** |  |  |  |  |  |
| Late endothelial failure | 45 | ref=1 |  |  |  |
| Allograft rejection | 109 | 0.830 | 0.587 | 0.423 | 1.628 |
| Primary graft failure | 44 | 0.723 | 0.425 | 0.326 | 1.604 |
| Pseudophakic bullous keratopathy | 25 | 1.015 | 0.973 | 0.413 | 2.495 |
| Corneal dystrophy | 17 | 0.176 | 0.096 | 0.023 | 1.357 |
| Post infectious scar/ thinning | 15 | 0.722 | 0.615 | 0.204 | 2.563 |
| Other causes of scarring / edema | 11 | 0.258 | 0.194 | 0.033 | 1.991 |
| Corneal injury | 6 | 0.594 | 0.617 | 0.077 | 4.574 |
| Glaucoma | 4 | 1.269 | 0.819 | 0.165 | 9.781 |
| Others | 8 | 0.789 | 0.756 | 0.176 | 3.532 |
| **Time between grafts (years)** | 284 | 0.938 | 0.030 | 0.886 | 0.994 |
| **Concurrent intraocular surgery** |  |  |  |  |  |
| Yes | 47 | 0.667 | 0.238 | 0.340 | 1.307 |
| No | 237 | ref=1 |  |  |  |
| **Regraft rejection** |  |  |  |  |  |
| Yes | 20 | 3.337 | <0.001 | 1.749 | 6.367 |
| No | 264 | ref=1 |  |  |  |

CI, confidence interval; ref, reference; PK, penetrating keratoplasty; DALK, deep anterior lamellar keratoplasty; EK, endothelial keratoplasty; IOP, intraocular pressure; FED, Fuchs endothelial dystrophy
