# Supplementary figures and images for: Ten-year outcomes of repeat keratoplasty for optical indications
Source: Front Med (Lausanne). 2025 Jan 22;11:1503333. doi: 10.3389/fmed.2024.1503333 (PMC11796611; doi:10.3389/fmed.2024.1503333)

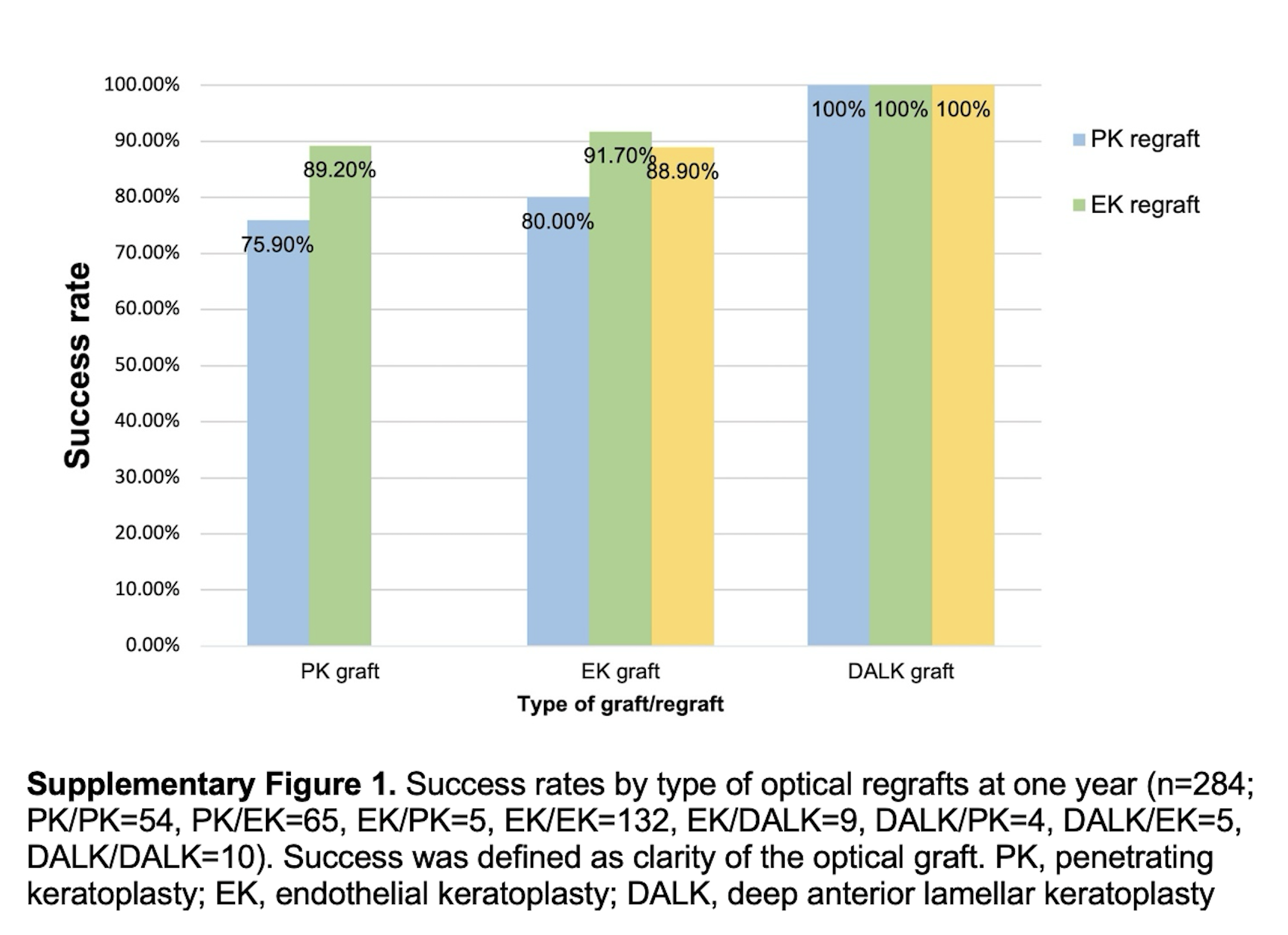

Supplement: Supplementary file 6 [file Image_1.png]
